# Heading north: Late Pleistocene environments and human dispersals in central and eastern Asia

**DOI:** 10.1371/journal.pone.0216433

**Published:** 2019-05-29

**Authors:** Feng Li, Nils Vanwezer, Nicole Boivin, Xing Gao, Florian Ott, Michael Petraglia, Patrick Roberts

**Affiliations:** 1 Key Laboratory of Vertebrate Evolution and Human Origins of Chinese Academy of Sciences, Institute of Vertebrate Paleontology and Paleoanthropology, Chinese Academy of Sciences, Beijing, China; 2 CAS Center for Excellence in Life and Paleoenvironment, Beijing, China; 3 Department of Archaeology, Max Planck Institute for the Science of Human History, Jena, Germany; 4 University of Chinese Academy of Sciences, Beijing, China; 5 Human Origins Program, National Museum of Natural History, Smithsonian Institution, Washington, D.C., United States of America; 6 School of Social Science, The University of Queensland, St Lucia, Brisbane, Australia; University at Buffalo - The State University of New York, UNITED STATES

## Abstract

The adaptability of our species, as revealed by the geographic routes and palaeoenvironmental contexts of human dispersal beyond Africa, is a prominent topic in archaeology and palaeoanthropology. Northern and Central Asia have largely been neglected as it has been assumed that the deserts and mountain ranges of these regions acted as ‘barriers’, forcing human populations to arc north into temperate and arctic Siberia. Here, we test this proposition by constructing Least Cost Path models of human dispersal under glacial and interstadial conditions between prominent archaeological sites in Central and East Asia. Incorporating information from palaeoclimatic, palaeolake, and archaeological data, we demonstrate that regions such as the Gobi Desert and the Altai Mountain chains could have periodically acted as corridors and routes for human dispersals and framing biological interactions between hominin populations. Review of the archaeological datasets in these regions indicates the necessity of wide-scale archaeological survey and excavations in many poorly documented parts of Eurasia. We argue that such work is likely to highlight the ‘northern routes’ of human dispersal as variable, yet crucial, foci for understanding the extreme adaptive plasticity characteristic of the emergence of *Homo sapiens* as a global species, as well as the cultural and biological hybridization of the diverse hominin species present in Asia during the Late Pleistocene.

## Introduction

Understanding the nature and tempo of Late Pleistocene dispersals of our species, *Homo sapiens*, across Eurasia is a critical issue in human evolutionary studies [1, 2]. Central and eastern Asia are playing an increasingly prominent role in palaeoanthropological investigations. Eastern Asia, and particularly China, has yielded some of the earliest fossil examples of our species anywhere in the world, *c*. 100ka in the southern, sub-tropical portion of the country [3–6]. Meanwhile, archaeological research in Central Asia, the Siberian Altai, and northern China, suggests that *Homo sapiens* only extended into and across this part of the world *c*. 45ka [7–11]. Recent chronological, archaeogenetic, and archaeological research at Denisova Cave in the Altai Mountains has provided high-resolution insights into the cultural and technological changes that followed the arrival of our species, and its interaction and eventual replacement of other hominin species, in northern and Central Asia [12, 13]. The Salkhit skullcap, which shows possible archaic features, has been subject to recent dating and mitochondrial DNA analysis, demonstrating that modern human populations were certainly present in Mongolia by *c*. 34ka [14]. The earliest modern human fossils found in North China are from Tianyuan Cave directly dated to 40ka [15] and the Zhoukoudian Upper Cave minimally dated to 35.1–33.5ka by a recently revised dating program [16]. Yet, despite the growth of detailed investigation of Late Pleistocene archaeological sites in Russia, Mongolia, and China over the past two decades, as well as their rich records of lithic and organic remains, there has been limited, detailed discussion of the temporal and spatial distribution of the potential routes of human migrations and their subsequent adaptations to a range of ecological settings.

**Fig 1:**
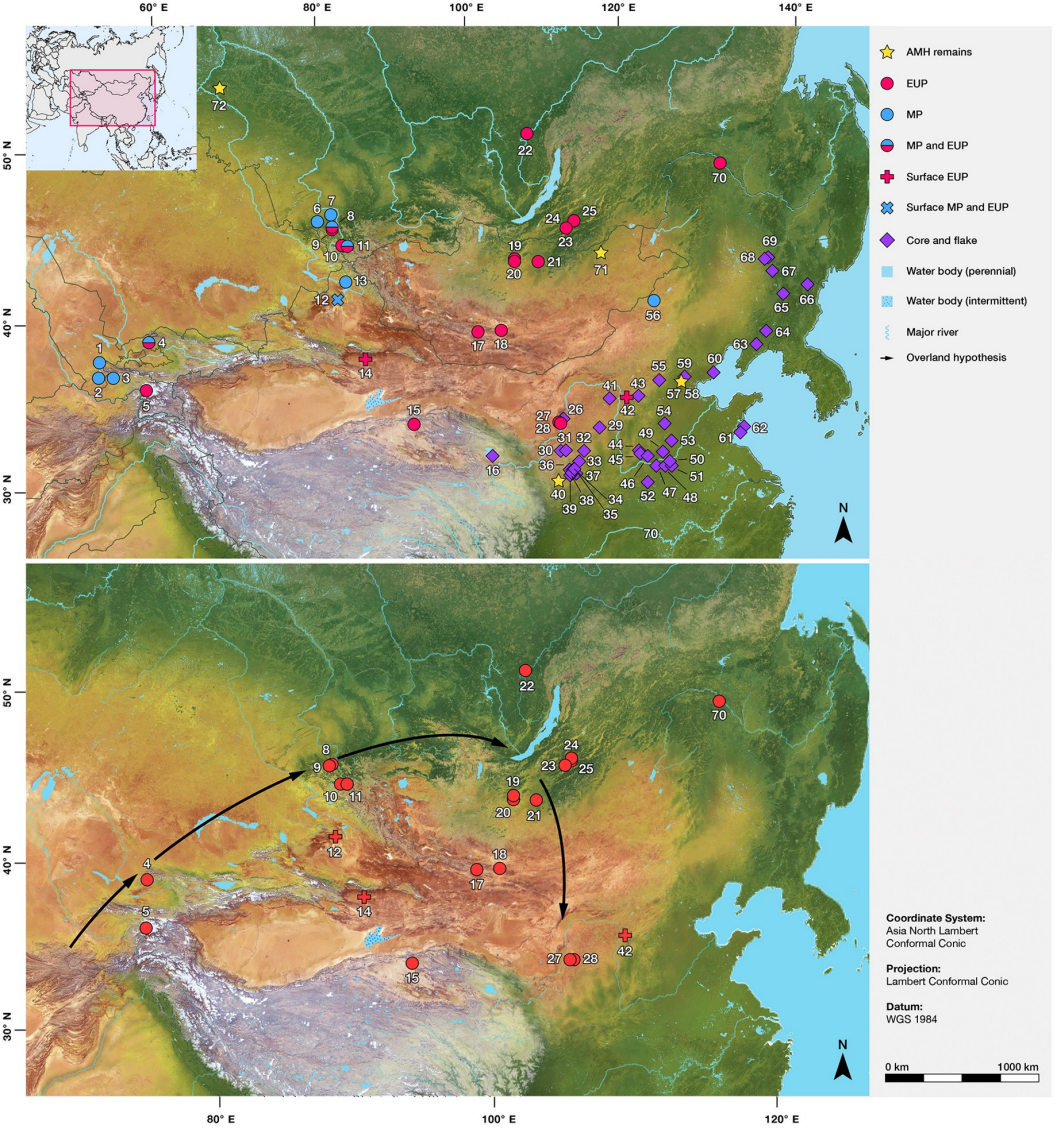
(A) Map of Initial Upper Palaeolithic (IUP), Middle Palaeolithic (MP) and core and flake archaeological sites, as well as sites containing *Homo sapiens* fossils across all the countries in Central and Northern Asia. (B) Map displaying the dispersal route suggested by Goebel’s [24] “Overland” hypothesis together with the all the known IUP sites in the region (S1 Table). Sites: 1. Anghilak cave, 2. Teshik Tash, 3. Khudji, 4. Obi-Rakhmat, 5. Shugnou, 6. Chagyrskaya, 7. Okladnikov, 8. Denisova, 9. Ust-Karakol, 10. Kara-Tenesh, 11. Kara-Bom, 12. Luotuoshi, 13. Tongtian Cave, 14. Gouxi, 15. Lenghu, 16. Heimahe 1, 17. Chikhen Agui, 18. Tsagaan Agui, 19. Tolbor 4, 20. Kharganyn Gol 5, 21. Orkhon 1 & 7, 22. Makarovo 4, 23. Kandabaevo, 24. Varvarina Gora, 25. Tolbaga, 26. Temple Canyon 1, 27. Shuidonggou 1, 27. Shuidonggou 2, 27. Shuidonggou 7, 28. Shuidonggou 9, 29. Fanjiagouwan (Salawusu), 29. Yangshugouwan (Salawusu), 30. TX08, 31. TX03, 32. Liujiacha, 33. GY03, 34. ZS08, 35. ZL05, 36. Shuangbuzi, 37. Shixiakou 2, 38. Xujiacheng, 39. Changweigou, 40. Gutougou, 41. Wulanmulun, 42. Yushuwan, 43. Shiyu, 44. Dingcun (7701), 45. Licunxigou, 46. Fuyihe (Xiachuan), 47. Beiyao, 48. Zhiji, 49. Tashuihe, 50. Laonainaimiao, 51. Huangdikou, 52. Longquandong, 53. Xiaonanhai, 54. Dangcheng (Shidie), 55. Xibamaying, 56. Jinsitai, 57. Tianyuandong, 58. Upper cave, 59. Wangfujing, 60. Zhuacun, 61. Huangniliang, 62. Dazhushan, 63. Xiaogushan, 64. Miaohoushan, 65. Xianrendong, 66. Shimenshan, 67. Zhoujiayoufang, 68. Yanjiagang, 69. Guxiangtun, 70. Shibazhan (75075), 71. Salkhit, 72. Ust’-Ishim. Base map raster is from naturalearthdata.com.

For many years, archaeological, biological, and genetic investigations have focused on the ‘southern’ routes of human dispersal throughout the Middle East and Asia [7–10]. *Homo sapiens* is suggested to have arrived in southern China, following expansion along the Indian Ocean rim [11, 17, 18]. The arrival of humans into the higher latitudes of Asia is seen as a much later phenomenon but has been relatively under-explored until the last few years [1, 19–21]. The main model in this regard suggests that the distribution of early ‘Upper Palaeolithic’ assemblages in northern Asia indicates that humans moved between central and eastern Asia by travelling north via the Siberian Altai, northern Mongolia, and the Transbaikal region before heading southwards into East Asia [22, 23] (Fig 1). The ‘Overland Model’, proposed by Goebel among others [24] is of great value as a working hypothesis, but this model assumes that west-east movements across the Altai and Tian Shan Mountains, and the modern Gobi and Taklamakan Desert region of southern Mongolia and northern China, were persistently too challenging. These zones are thought to represent cold, high-altitude, and dry desert ‘barriers’, leading archaeologists to largely neglect them in terms of survey and their potential for being important routes of human dispersal.

The last two decades of research into Late Pleistocene (125-12ka) human dispersals has, however, revealed that modern arid environments, such as those found in the Arabian peninsula and the Thar Desert of northwestern India, as well as palaeoarctic and high-altitude regions, traditionally assumed to be dispersal barriers [9, 25–27], were passable at certain periods in the past [10, 28–30]. Palaeoclimatic and terrain modeling research has confirmed the appearance of periodic pathways, while new archaeological research has detailed significant hominin presence in the past [9, 25, 28–30]. Moreover, it is being increasingly appreciated that our species documents a unique adaptive plasticity in the face of variable habitats and climates when compared to other hominins [31–34]. As a result, it seems likely that renewed investigation and appraisal of the region covered by the varied environments of the Gobi Desert, the Tian Shan, and Altai Mountains, spanning Uzbekistan into northern China, could reveal new potential routes of human dispersal between central and eastern Asia. If this is the case, this would highlight the need to investigate new regions in order to examine the unique ecological capacities of our species and the degree of interaction between *Homo sapiens*, *Homo neanderthalensis*, and Denisovans.

Here, we use Geographic Information Systems (GIS) software, alongside the most detailed palaeoclimatic, palaeoglacier, and palaeolake datasets available, to investigate the possible routes of human dispersals between Central and eastern Asia under postulated glacial and interstadial conditions between MIS5 and MIS3. In building the first Least Cost Path models for human dispersals across this part of Asia, we follow the work performed by Field and Lahr [35] and Field et al. [7] for southern dispersal routes and Kealy et al. [36] for corridors extending across Sunda to Sahul. Existing dispersal models for modern human expansions across Eurasia link movements to lithic technocomplexes classed variously as ‘Upper Palaeolithic’, ‘Early Upper Palaeolithic’, and ‘Initial Upper Palaeolithic’ [37–40]. There has been an almost complete lack of systematic overview of the changing potential topographic, climatic, and environmental constraints of the regions studied. Therefore, we seek to provide a new perspective in this regard by producing models that visualize the summary of the effects these variables would have had on human population movements at different points in time, as well as a means of recommending key locations in Mongolia and China for future archaeological survey and excavation.

## Methods

### GIS models

A GIS approach provides a computational understanding of the best routes to travel across the vast region of Central, northern, and eastern Asia. Specifically, this GIS approach looks to integrate archaeological, environmental, and geographic data in a spatial manner. The Least Cost Path analysis method in ArcGIS 10.5.1 provides an analysis of the route between two points across a rasterized cost surface terrain. Although it has been used extensively in ecology to explore the dispersal corridors and migration routes of animals [41, 42], only a few macro-scale archaeological studies have employed this method in a Late Pleistocene context [7, 35, 36, 43].

Two palaeogeographic maps were produced as a base for the Least Cost Path. One simulated a dry ‘glacial’ climate using precipitation data from WorldClim’s downscaled Last Glacial Maximum (LGM) simulation from the Community Climate System Model 4.0 (CCSM4). We use the LGM model as it provides the best, most complete framework for estimating the climatic impacts of glacial conditions during MIS4 for the region [44]. The other model simulated ‘interstadial’ conditions but, due to a lack of suitably detailed precipitation models for MIS5 and MIS3 in the region we focused on relevant, dated topographic and geographic features that may have influenced dispersal during these periods (see below). As a working hypothesis, we assume that the presence of these features, as well as palaeoenvironmental datasets (see below), indicates that aridity did not act as a barrier in these periods.

Dates from the region suggest the earliest IUP appears during MIS3. These maps provide the ‘cost surfaces’ for the Least Cost Path analysis to determine the most effective route of this arrival. To create these cost surfaces, we first assembled spatial palaeolandscapes using topographic data overlain with modern and published palaeolake extents for the MIS3 model and LGM glacier extents and LGM precipitation estimates for the ‘glacial’ model.

The base topographic map used for both models was the HYDRO1K digital elevation model that has a resolution of 30 arc-seconds (~1km^2^). The slope of the map was taken to calculate the cost (S2 Table) and reclassified to a scale that better reflects terrain difficulty costs for humans based on the assumption that steeper slopes are more difficult to traverse [45]. To simulate the LGM, regions with an average annual precipitation of less than <250mm, based on world climate models of the LGM, were treated as barriers, and given an appropriate value according to Field and Lahr [35] that would allow short movements across these regions; we believe this would mirror the ability for populations to traverse arid regions [7].

Published MIS5-MIS3 palaeolake extents were obtained from a variety of sources (S3 Table), and were outlined and downscaled to a 1km resolution. A number of geographical investigations into northern Asian palaeolakes suggest large MIS5 lake extents. Many of these were originally radiocarbon dated to MIS3 using radiocarbon methods, but recent OSL dating of the alluvial sediment suggests they could also date to MIS5. Despite the re-dating, some demonstrate high lake stands, but further analysis and discussion is still warranted. Therefore, we used the MIS5 lake stands as a proxy for the wet climate of interstadials between MIS5 and MIS3, potentially enabling hominin dispersal across these arid regions, but also as geographical barriers due to their large size. It is hoped that in the future, more detailed climate simulations of precipitation for MIS3 and MIS5 will enable the production of more comprehensive interstadial base maps for more comprehensive analyses for these periods in the future.

Available raster files of LGM glacial extents from the collection of data from Ehlers et al.’s [46] LGM extents for Asia and Kuhle's [47] LGM extents for the Tian Shan were also converted into a 1km^2^ raster. The local LGM of northern Asia is around 28-15ka. Although the volume of ice may not have been so large in MIS3, studies [48] have shown that their geographic extent may have been equal to those of the LGM. Hence, the extents of the glaciers of northern Asia are used as barriers for *both* the wet and dry models [48, 49]. In addition, similar to Field and Lahr [35] we added both modern lakes and rivers [50, 51] as barriers to both models, but with the condition that only river widths larger than 1km were considered impassable. All the impermeable barriers were given the value of *NoData*. Finally, all of these geographic factors were combined with the slope map to create a base palaeogeographic cost surface for the wet and dry models. The dry model was given the conditions of aridity described above, as an additional barrier, limiting colonization of specific regions with a lack of water (see S1 and S2 Figs for the cost surfaces and cost distance of the wet and dry models, and S2 Table for costs).

### Setting the origins of the model and archaeological comparison

**Fig 2:**
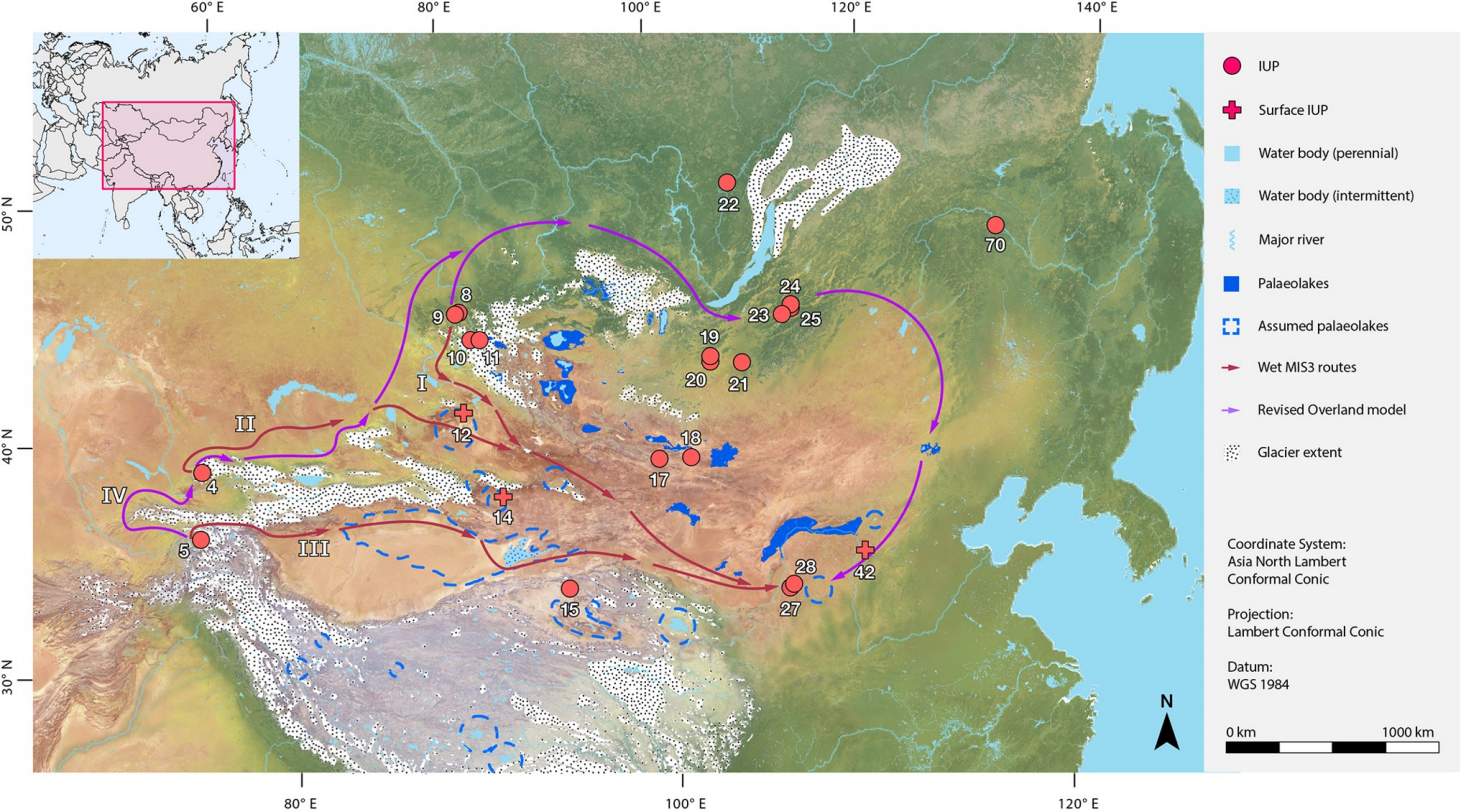
Illustrated dispersal routes from the results of the Least Cost Path analysis. The three routes from the “wet” simulations and the single route from the “dry” simulation are presented together in conjunction with palaeoclimatic extents (glaciers and palaeolakes). Sites: 4. Obi-Rakhmat, 5. Shugnou, 8. Denisova, 9. Ust-Karakol, 10. Kara-Tenesh, 11. Kara-Bom, 12. Luotuoshi, 14. Gouxi, 15. Lenghu 1, 17. Chikhen Agui, 18. Tsagaan Agui, 19. Tolbor 4, 20. Kharganyn Gol 5, 21. Orkhon 1 & 7, 22. Makarovo 4, 23. Kandabaevo, 24. Varvarina Gora, 25. Tolbaga, 27. Shuidonggou 1, 28. Shuidonggou 9, 42. Yushuwan, 70. Shibazhan (75075). I. ‘Altai’ Route, II. ‘Tian Shan’ Route, III. ‘Tarim’ Route, IV. “Revised Overland’ Route. Base map raster is from naturalearthdata.com.

ArcGIS’s cost distance tool was used to create cumulative cost raster maps from three possible dispersal centers: the Altai Mountains, the Tian Shan Mountains, and the Pamir Mountains (Fig 2, See S1 Table for information on the sites). The site of Denisova was used as the arbitrary coordinate for the Altai (51.2°N, 84.7°E), which has IUP assemblages dated up to ~49ka [13], and several sites in the vicinity, such as Ust-Karakol, Kara-Tenesh and Kara-Bom [52] all suggest that the Altai may have been a center of a population large enough to encourage dispersals. Similarly, Obi Rakhmat (41.567°N, 70.133°E) in the Tian Shan Mountains, has a large IUP assemblage and it is one of the few stratified sites in the region, and it was therefore selected to be the of the dispersal origin point for this region [53, 54]. Shugnou (38.481°N, 71.097°E), in the Pamirs, was selected as the third origin point as it too is one of the few stratified sites with IUP assemblages in that region [55]. The destination point for the production of all three maps was the Shuidonggou site (38.377°N, 106.512°E), as this is the easternmost known site with IUP material [38]. We are, of course, not suggesting that all humans dispersing east during MIS3 arrived at Shuidonggou, but that to our current knowledge it is the furthest East that IUP populations reached in northeast Asia. The origins and destination points of the Least Cost Path remained the same for the dry and wet models.

Lithic assemblages were categorized into three major categories: Middle Palaeolithic (MP) with typical Levallois reduction and tool typology, Initial Upper Palaeolithic (IUP) with tool forms and blades produced by a combination of Levallois and volumetric prismatic methods, and core-flake technocomplex typified by more simply-organized flake reduction and lightly retouched tools (Fig 1). The most recent dating program at Denisova Cave convincingly associates the Middle Palaeolithic with Neanderthals (193-97ka) and Denisovans (287-55ka) [56]. Recent dating of ornaments and the IUP bearing layers at Denisova cannot yet be attributed to a species, although the age range corresponds with the age of the *Homo sapiens* fossil at Ust-Ishim [19]. In addition, increasingly frequent use of ornaments, pigments, and formal bone tools is also associated with IUP assemblages in Siberia and Central Asia [52, 57], and so far such finds are not found in MP sites.

Though the *Homo sapiens* fossil from Salkhit is not directly associated with archaeological finds, the 34ka age is argued to correspond with Levallois and blade assemblages (or the ‘early Upper Palaeolithic’) in Mongolia [14]. Therefore, we maintain that the spatial distribution of the IUP likely associates with the widespread dispersal of *Homo sapiens* populations across North Asia during MIS 3 [39, 40], notwithstanding the possibility for interbreeding with indigenous archaic populations. Accordingly, we compiled and mapped the available, well-dated instances of the main technocomplexes for Russian Siberia, Mongolia, and North China. Sites from several other central Asian countries, including Tajikistan and Uzbekistan, representing major potential areas of human dispersal, are also included to provide good comparisons of route trajectories to known archaeological material.

### Selection of climatic records for comparison with model assumptions

**Fig 3:**
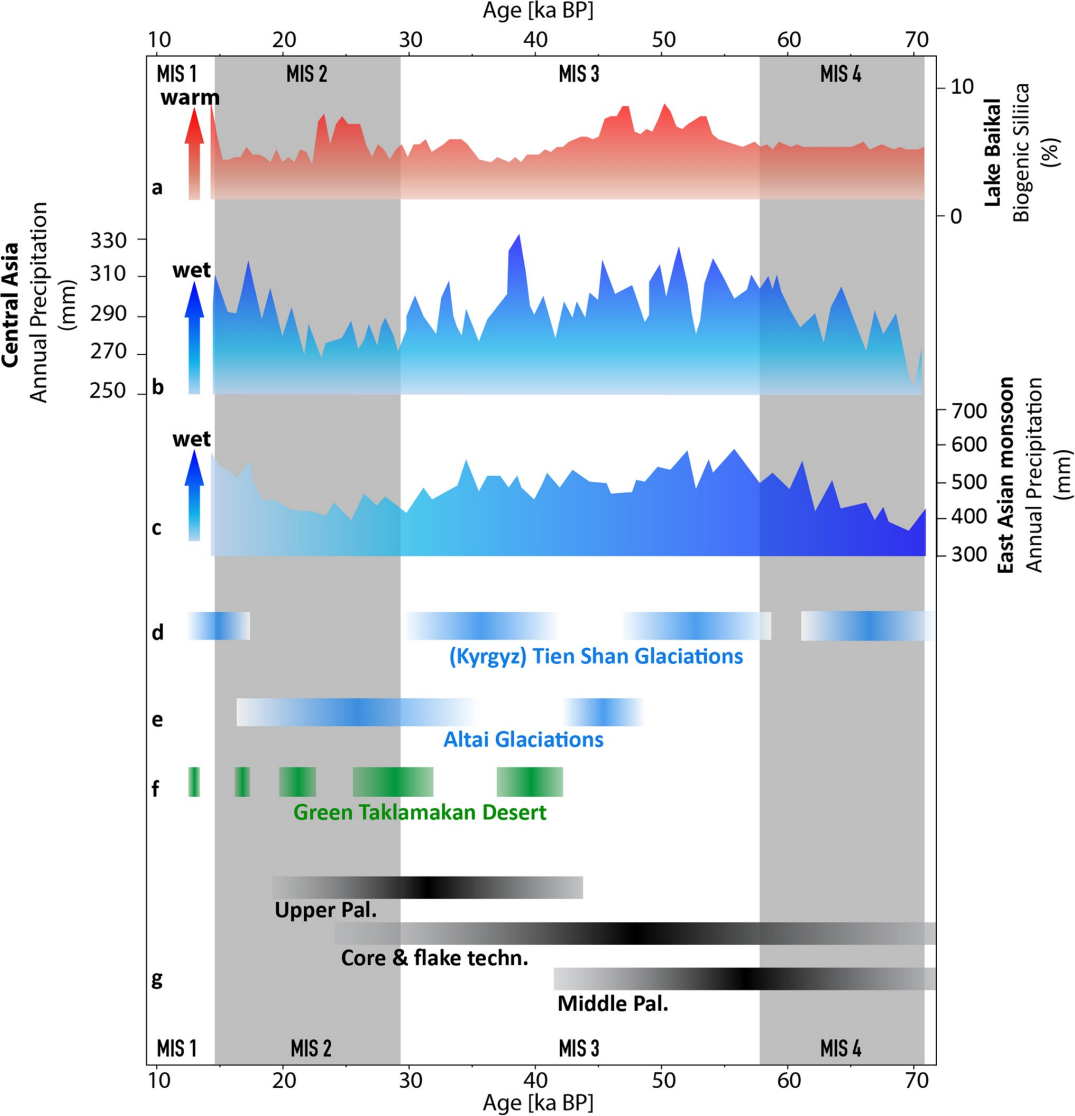
Comparison of climatic records and archaeology spanning the past 70,000 years. (A) Orbitally tuned Lake Baikal biogenic silica record as a relative temperature proxy, due to insolation forcing [48]. (B,C) Modeled annual precipitation for Central Asia (B) and the East Asian Monsoon (C) [38]. (D,E) Timing of glacier advances for the Kyrgyz Tian Shan (D) [76] and the Altai (E) [50]. (F) Timing of wetter periods for the region of the Taklamakan Desert [51]. (G) Timing of archeological technologies grouped into three major categories: Middle Paleolithic (MP), Initial Upper Paleolithic (IUP), and the core-flake technocomplex.

The climatic records shown in Fig 3 provide a chronological overview of environmental changes across the region to act as a more temporally resolved, detailed points of comparison for the periods under discussion. The environmental data sources selected are 1) all archives representing terrestrial regions, 2) all located within the study area, and 3) cover the Late Pleistocene period from at least 70ka to 12ka. The Lake Baikal biogenic silica dataset represents a relative temperature record for southeastern Siberia. The dataset has been orbitally tuned and suggests that maxima in biogenic silica content (amount of diatoms) coincide with insolation peaks. Hence, the warmest periods during interglacial conditions can be directly inferred from high biogenic silica contents [58].

The precipitation records for Central Asia (CA) and the East Asian Monsoon (EAM) shown in Fig 3 have been simulated using the Community Climate System Model version 3. The spatial coverage for the EAM and CA precipitation regimes are 100°E-120°E, 35°N–50°N and 50°E–70°E, 35°N–50°N, respectively [59]. Reconstructed glacier advances in the Kyrgyz Tian Shan and the Altai rely on surface exposure dating of moraine complexes. The displayed Kyrgyz Tian Shan and the Altai data comprise the mountain ranges of the Kyrgyz Front in the north, the Terskey Ala Tau in the east, the At Bashi to the south of the Kyrgyz Tian Shan [49], and the Ikh Turgen Mountains in the Altai [60]. Glacier advances are linked to increasing precipitation.

The greening of the Taklamakan Desert shown in Fig 3 is based on the investigation of lacustrine sediments of terminal lakes in the Taklamakan Desert; these lakes were re-activated by the inflow of perennial dry-land rivers. It is argued that the latter could have resulted from intensified westerlies and/or increasing glacier melting affecting in particular the desert margins [61]. Together, these palaeoaenvironmental features were used to test the assumption that aridity would not have been a barrier to human movements in this part of the world during MIS5 and MIS3 interstadials, as per our interstadial model. Similarly, they enable us to further test to the degree to which aridity would have limited movement across Central Asia under glacial conditions in MIS4.

## Results

### ‘Glacial’ conditions

The Least Cost Path analysis simulation under ‘dry’ conditions (i.e. using Last Glacial Maximum (LGM) climate models produced a ‘Revised Overland’ Route that we expect to hold for conditions during both MIS4 and MIS2 (Fig 2, S3 Fig). The same trajectory Least Cost Path towards Shuidonggou was followed regardless which of the three starting point sites were used. The result largely coincides with the prevalent, previously-published Overland Route [24]. However, the Least Cost Path model produced here integrates Siberian topography and palaeoclimatic barriers to produce a more detailed model of potential human movement. The beginning of this route skirts the westernmost piedmonts of the Gissar Range of the Tian Shan Mountains, rather than directly moving eastwards through river valleys. Upon reaching the piedmonts of the Talas Alatau Range, the Least Cost Path moves east along the range, being channeled between the Karatau Mountains. At the Dzungarian Alatau it continues between Lake Balkash and Lake Alakol through to the Tabagatay Range before reaching the western edge of the Altai Mountains and crossing the Irtysh River.

Moving northeast, the route rounds the Altai Mountains and moves into the Siberian Plain, heading towards the Yeniseri River. From this point onwards, the route continues into the plains adjacent to the Eastern Sayan Mountains, before passing the edge of Lake Baikal and the mouth of the Angara River. As the route passes to the eastern side of Lake Baikal, it travels through a number of valleys of the Reka Ingoda River, which channel the route further east than Shuidonggou itself. The Least Cost Path turns south upon reaching Julun Lake and the Ergon River, passing the Buir and Dalinur Lakes before reaching the Taihang Mountains. The ‘Revised Overland’ Route then curves west before crossing the Yellow River and travelling parallel to the Jilantai Basin before it finally reaches the site of Shuidonggou.

One of the main factors driving this route to the north, as in the case of the original Overland Route, is the hyper-aridity throughout Central Asia during glacial periods also observed in a number of climate records [44] (Fig 3). As such, the route is driven along a relatively mountainous pathway, with short distances travelled across arid regions, such as the basin of the Tarbagatay Range and the Trans-Baikal. Within this mountainous context, LGM glacial extents present additional challenges, meaning that many valleys were likely inaccessible to hominin groups. The route is therefore dominated by mountain piedmonts, with only one glacial corridor used, that between the northern Tian Shan and Karatay Mountains. In Siberia, the glaciers northwest of Lake Baikal also force the route along the plains of the region, away from potential mountain routes. As this is a dry period, palaeolakes play a minor role in shaping the trajectory of this Least Cost Path. However, palaeolakes at Jinpeng-Dali Nor [62], Daihai Lake [63], Jilantai-Hetao Lake [64], and Salawusu [65] may have represented key sources of water and resources towards the end of this route.

### ‘Interstadial’ conditions

Three Least Cost Path routes were the product of the simulation of “wet routes” (i.e., under postulated wet MIS5 and MIS3 conditions) (S3 Fig). We have named these routes the ‘Altai’ Route (Route I), the ‘Tian Shan’ Route (Route II), and the ‘Tarim’ Route (Route III), respectively. The ‘Altai’ Route simulates dispersal between the Russian Altai Mountains (using the site of Denisova as the center point) towards the Yellow River valley and the location of the Shuidonggou site in China. The ‘Tian Shan’ Route originates in the western Tian Shan Mountains, while the ‘Tarim’ Route demonstrates the best route from the Northwestern Pamir Mountains (Fig 2).

### Route I: ‘Altai’ Route

**Fig 4:**
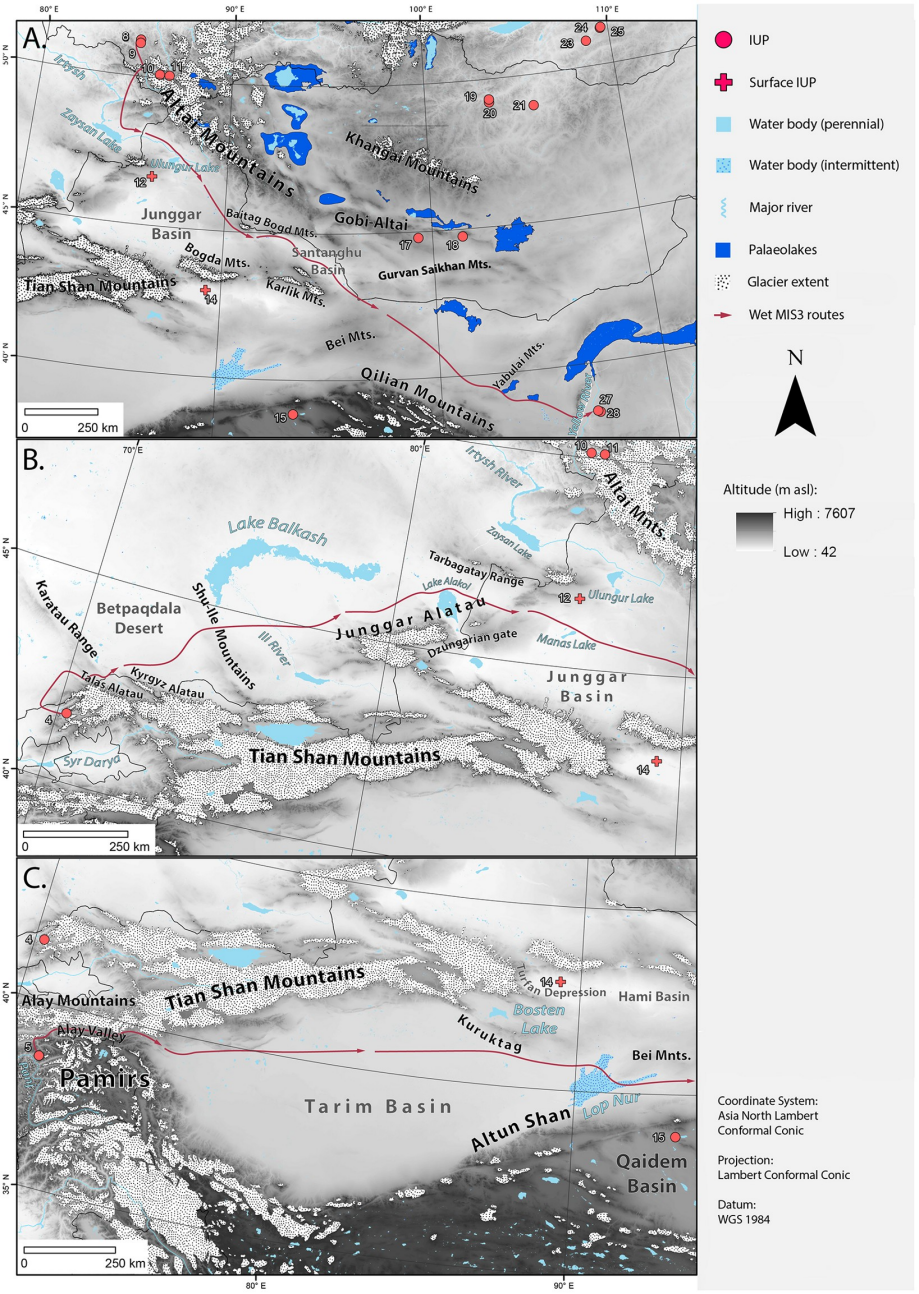
(A) Map of geographic features that guide or border the ‘Altai’ Route. (B) Map of geographic features that guide or border the ‘Tian Shan’ Route. The eastern part of the route after the Junggar Basin connects with the ‘Altai’ Route. (C) Map of geographic features that guide or border the ‘Tarim’ Route. The eastern part of the route after the Bei Mountains connects with the two other routes. Sites: 4. Obi-Rakhmat, 5. Shugnou, 8. Denisova, 9. Ust-Karakol, 10. Kara-Tenesh, 11. Kara-Bom, 12. Luotuoshi, 14. Gouxi, 15. Lenghu 1, 17. Chikhen Agui, 18. Tsagaan Agui, 19. Tolbor 4, 20. Kharganyn Gol 5, 21. Orkhon 1 & 7, 22. Makarovo 4, 23. Kandabaevo, 24. Varvarina Gora, 25. Tolbaga, 27. Shuidonggou 1, 28. Shuidonggou 9. Base map is HYDRO1K.

This route begins by following the modern day Anuy Valley before joining the tributaries of the Charysh River. None of the rivers are large enough water bodies to be considered barriers to movement in our model. The Least Cost Path then descends into the catchment of the Irtysh River of Kazakhstan (Fig 4A). Once it reaches an impassable width of the river and Zaysan Lake, it skirts the southern end of the Altai Mountain chain before crossing a passable section of the Irtysh and entering northern China and the Junggar Basin. Staying between the Altai Mountains and the Ulungur and Jili lakes, the path descends towards the southeast of the basin and enters the Gobi-Altai region. Before exiting the Junggar Basin, the route passes between the Bogda and Baitag Bogd Mountains. Eventually it passes through the Santanghu Basin between the terminations of the Tian Shan, the Karlik Mountains, as well as the Gobi-Altai, and Gurvan Saikhan Mountains. The Least Cost Path finally curves south to skirt along the boundaries of the northern rim of the Bei and then the Qilian Mountains, traversing through the Yabulai Mountains before ending in the valley of the Yellow River.

In addition to the basic topographic constraints, certain palaeoclimatic barriers guide the ‘Altai’ Route. For example, glacier extents during the LGM [46] (Fig 2) were at their largest, which had increased since MIS4 and MIS3 [48, 60] implying that ice and glacial dammed lakes, such as at Kanas River [66], Hoton Nuur and the Chuja Basin, would have covered the majority of the Altai Central Mountain complex [67, 68]. As these features would have been largely impassable, these features force the model to bypass around to the south. Glacial features also prevent this route ‘contacting’ the large palaeolake water bodies in Mongolia. Towards the end of the route, the palaeolakes of Balikun [69], the Ejina Basin [70], and Yabrai and Zhuye [71], would have represented potential corridors for movement. Indeed, the Jilantai-Hetao megalake [64], would have provided a key final conduit for final movement through a relatively arid region to the site of Shuidonggou.

A lack of archaeological survey and excavation along much of this route means that confirming humans may have taken such a path will require future work. At the beginning of the route, there are a series of significant Late Pleistocene sites in the Altai region, including Denisova cave, Kara-Tesh, Kara Bom, and Ust-Karakol [72, 73]. All of these sites have IUP lithic material in them, and some, such as Kara Bom and Denisova, also possess Middle Palaeolithic artefacts [3]. Overall, however, there is little known IUP archaeological evidence along most of the remainder of the Altai Route. One exception is the surface site of Luotuoshi [72], which contains typologically IUP assemblages, and is situated close to Manas Lake. Two additional archaeological sites, Chikhen Agui and Tsaagan Agui, within 400-600km of the Least Cost Path, and on the Altai Mountain chain, have IUP lithic assemblages [38, 73, 74].

### Route II: ‘Tian Shan’ Route

This route begins along the Charvak Basin of the Talas Alatau range (Fig 4B). Its topographic path then descends along the Chirchiq River Valley, eventually crossing the river itself before curving northeast around the Talas Alatau. The Least Cost Path is constrained between the Karatau Mountains and the Kyrgyz Alatau before crossing the modern day Betpaqdala Desert. Continuing east, this model crosses the Ili River, south of Lake Balkhash and east of the Junggar Alatau. Much of this region is flat with limited elevation changes leading to a linear trajectory towards, and then around, the Junggar Alatau. At the western edge of Lake Alakol the pathway shifts to the north of this water body before heading through the valley between the Tarbagatay Range and Junggar Alatau, avoiding the Dzungarian Gate. When the model enters the Junggar Basin and reaches the Ailike and Manas Lakes, it takes a linear route to Shuiddongou. It is at this point, where the Chinese provinces of Xinjiang and Inner Mongolia intersect with Mongolia, that this model meets and follows the Altai Route.

In the context of palaeoclimatic features, the direction of the model is reinforced by the fact that the steep slopes of the high altitude Tian Shan Mountains would have been glaciated through the Late Pleistocene, with an equilibrium line (ELA) of ~1300m a.s.l [47] (Fig 4). Similarly, glaciation extents within the Dzungarian Alatau would have further encouraged the valley trajectories of the Tian Shan Route described here. The initial part of this route only passes one potential palaeolake at Manas Lake [75]. While no published MIS3 extents of this lake are currently available, the Least Cost Path does pass by this water body, and would have its direction modified should the lake have been larger during the Late Pleistocene. The majority of the Dzungarian Basin covered by this route is currently desert or hyper-arid, suggesting that most modern lakes are a fraction of the size that they might have been at various points in the past.

### Route III: ‘Tarim’ Route

The final ‘wet route’ model produced a path from the northeast side of the Pamir Mountains in modern day Tajikistan, to the destination of Shuidonggou. As with the previous two routes, the ‘‘Tarim’ Route’ begins in a mountain valley, that of the Panj tributary of the Amu Darya (Fig 4C). However, this route begins at a much higher elevation (2000 m a.s.l) compared to Routes I and II, continuing into the northern Pamir Mountains via the Obikhingou River before joining and following the Kyzylsu River upstream. Taking the route of this river eastwards, this route bisects, and then continues along, the Vaksh River before it reaches the large Alay Valley, which acts as a major conduit. The Least Cost Path then descends into the Tarim Basin, linearly moving east, close to the Tian Shan Mountains, without any significant changes in elevation through the Taklamakan Desert. The route skirts around the southern side of the Kuruktag, before going through the current Lop Nur Salt flats. Reaching the northern side of the Altun Mountains, it passes the Qaidem Basin before squeezing through the Bei and the Qilian Mountains. It then joins the ‘Altai’ and ‘Tian Shan’ Routes 200km before Shuidonggou in the Yellow River Basin.

The ‘Tarim’ Route would have been reliant on corridors formed during certain palaeoclimatic conditions. The Alay Valley, and the smaller valleys of the Pamir range, would have been free of glaciers during the Late Pleistocene [59], providing an expansive corridor between the glaciers of the Tian Shan and the Tibetan Plateau, and potentially directing hominin expansion towards the Tarim Basin. Based on existing palaeoenvironmental proxy data, the palaeolake at Lop Nur would have covered the majority of the Tarim Basin, albeit with potentially significant fluctuations in its extent through time [77]. While there are currently many rivers in this Basin, the palaeolake would have dominated the landscape, shaping movement pathways but also providing a crucial water, and likely also biodiversity, source within the arid region. A large extended palaeolake (combining Chaerhan and Dachadan Lake) would also have existed *c*. 400km away in the Qaidem Basin [78, 79], while the palaeolakes of the Yabrai and Zhuye Lakes [71] would have represented similar opportunities towards the end of the route.

The ‘Tarim’ Route is located in proximity to a few interspersed IUP sites. It begins at Shugnou, which has little published on the IUP, but it is located at an important crossroads linking West and South Asia to Central Asia [55]. As the route nears the end of the Taklamakan Desert, it passes close to the IUP surface site of Gouxi in the current province of Xinjiang in China [80]. The final site before reaching the end of the route is Lenghu 1 [81], which lies just within the Qaidem Basin, 1000m above the Tarim Basin.

## Discussion

### From west to east?: New models of human movement between central and eastern Asia

The dispersal of *Homo sapiens*, or IUP producers, into Central, northern, and eastern Asia, has tended to be uniformly depicted as following a ‘northern’ route that extends around the Altai to the north, across Siberia and the Transbaikal, before turning southward into central and eastern China [22, 23, 37, 39, 40]. During ‘dry’ and ‘cold’ periods, the trajectory of the northern route is the only probable option, fitting with existing assumptions that passes across the Altai and Tian Shan Mountains, and routes across the Gobi and Taklamakan Deserts, would, at least periodically, have represented major barriers to hominin movements. Such a view is consistent with the work of Beeton and colleagues [82] and Glantz and colleagues [83] which observed a tendency for Middle and Late Pleistocene hominin populations to stick to montane regions during dry periods in Central Asia, as a result of their higher precipitation rates and reliable access to freshwater sources [49, 84] (S4 Fig). The Altai Mountains and Siberian Plains currently have much higher precipitation rates compared to the basins along the Tian Shan and Gobi Altai, suggesting they may have been particularly important refuges for human populations under dry, cold conditions.

However, our Least Cost Path models highlight that under wetter, warmer conditions direct west-east human population movements would have been possible across the Gobi and Taklamakan Deserts, and across the Altai and Tian Shan Mountain chains (S4 Fig). A number of archaeological sites are located close to palaeolakes with high MIS3 water levels, and the surrounding regions show lower levels of aridity throughout the whole of northern Asia. Though erosion and sand dunes may have obliterated and buried signs of palaeolake extents along the ‘Tarim’ and ‘Tian Shan’ Routes, wet periods are recorded in a number of places. At 40-30ka, lake deposits are found 100-200m above the current surface [61]. Palaeolakes around the Qaidem Basin date to MIS3 by radiocarbon and to MIS5 by OSL, and although ages are discrepant, they illustrate large changes to one of the most arid regions (24-40mm/a) in the world, with lake levels 60-160m higher than today [76]. To the east, the Zhuye Lake, Yabulai Salt Lake and Jilantai Salt Lake show higher lake levels during 37-23ka, 29-21ka and 37-23ka respectively. Additionally, OSL dating at Jilantai indicates even higher lake levels between 100-40ka [71]. Along the northern border of the Tian Shan Mountains, Manas Lake had 26m high lake levels at 80ka and 20m high lake levels at 66ka and 38ka [75].

Further east, uranium-series dating of Balikun shows high lake stands at 138-132ka, 69-65ka and 25-18ka [85]. Many of these lakes coincide with MIS3 lake stands and indicate lower aridity. While survey and chronological information remains limited, surface IUP sites are also found in the general region of the palaeolakes and river systems that may have sustained these routes during wetter periods, for example with a more active East Asian Monsoon. Little available survey data and lack of chronological constraints across the region currently make it difficult to pinpoint occupation of these surface sites to a specific ‘wet period’. Many of the surface sites are situated near lakes apparently during periods of higher lake levels, as seen, for example, with Tsagaan Agui’s [74] proximity to the Boontsagaan-Orog Nuur Palaeolake [86]. A number of the lakes that have been dated to MIS3 [43, 74, 87] may also have been present during MIS5, suggesting there could be several pluvial periods in the region (S5 Fig). The presence of palaeolakes in these regions offers testament to prehistoric wet phases and should stimulate future archaeological survey in the region. It is reasonable to assume that freshwater lakes and vegetation would have drawn in mammals and hunter-gatherer populations during ameliorated conditions in the Pleistocene, including *Homo sapiens* in the Late Pleistocene.

### Archaeological confirmation?

The results of our models do not definitively demonstrate west to east routes of human dispersal. However, they, alongside the growing palaeoenvironmental literature for the region (Fig 3; S5 Fig), do suggest their potential, something that has not been properly addressed in research about hominin movements between central and eastern Asia during the Late Pleistocene, and perhaps, the Middle Pleistocene. Archaeological evidence for Central and eastern Asia between MIS5-MIS2 broadly suggests a replacement of MP technologies by new IUP technologies from ~50ka, linked with the expansion of *Homo sapiens* populations (Fig 1). The routes and adaptations of these expanding human populations have major implications for understanding the relationship between our species and its hominin relatives [13, 88], as well as the novel ecological capacities exhibited by *Homo sapien*s [2, 34, 89]. Nevertheless, such discussions have frequently been based on assumption, with archaeological fieldwork following ‘likely’ regions of habitability to the neglect of more extreme desert and high altitude settings. As a result, existing archaeological sites in Russia, Mongolia, and China have been argued to confirm a relatively rapid ‘Overland’ human route north from the Altai Mountains, into the Siberian Trans-Baikal, and down into northeastern China taken between 50 and 45ka.

Certainly, during dry periods, our models, and the palaeoenvironmental literature support the Overland Route as a viable corridor of movement. This route is reinforced by a wealth of IUP sites along its trajectory. In particular, the identification of numerous sites around the Lake Baikal region, owing to much archaeological exploration, has driven support for the eastern extent of the Overland Model, connecting the lake with sites in the Tian Shan and Altai regions. The Tian Shan lacks any well dated sites besides those revisited in Fitzsimmons et al. [90]. These Trans-Baikal IUP sites are Varvina Gora, Kandabaevo, Tolbaga, with the earliest material dating to ~41ka, 43ka and 34ka respectively [91, 92]. In addition, the sites 500km to the south in the Selenge River valley, which feeds into Lake Baikal, of Tolbor 4, Orkhon 1 & 7, and Kharganyn Gol 5 have similarly early dates of 41ka, 44ka and 48ka, respectively [93–96]. In proximity to Shuidonggou, the path also passes the surface IUP site of Yushuwan [97, 98]. Whether the concentration of the sites of these regions are a reflection of past habitation patterns or simply preservation bias due to increasing aridity and erosion of the landscape in much of northern Asia is something worthy of further investigation.

The ‘Tarim’ Route suggests that increased precipitation and higher temperatures over Central Asia could have facilitated other, more direct dispersal routes into eastern Asia. For example, the earliest signs of human occupation in the Tarim Basin, dated to ~7ka, have been related to the presence of humid environments across the region [99], and point clearly to the possibility of prior expansion into the region during similar wet phases. Indeed, there is evidence for a number of periods of climatic amelioration within the Tarim Basin [61] (Fig 3). Thus, within hydrological favorable periods the Taklamakan Desert may have been a “green” corridor for a West-East traverse, similar to those conditions identified in the Sahara and Arabia [29, 100, 101]. Unfortunately, a lack of archaeological survey and excavation means that similar movements in MIS3 (or perhaps also MIS5) remain a possibility rather than identified reality. The route could have also passed the palaeolake of Lop Nur. The surface site of Luotuoshi, although undated [72], is located close to Manas Lake. Similarly, another IUP surface site, Gouxi is located near the palaeolake of Aiding palaeolake, where sediments indicate higher precipitation. Otherwise there is a lack of published surface sites in the region prevent proper examination of these environments bordering the lakes. Research in other regions such as Saudi Arabia has shown this to be the case. Along the ‘Tian Shan’ Route, the site of Obi-Rakhmat is one of the best-documented IUP sites in the Central Asian region [53, 54]. It is also one of the few archaeological sites currently known along this entire route. The other is the previously mentioned surface site of Luotuoshi, located in modern day Xinjiang [72]. The ‘Altai’ Route traverses much of the Altai Mountains glaciers as its origins are nestled within piedmonts. During MIS3 these glaciers may have been slightly smaller in their extent [102], allowing for more potential routes through the piedmonts, but not digitized extents exist. Many of its palaeolakes and archaeological sites that it passes are similar to those in the ‘Tian Shan’ Route.

### Conclusions: A new region in which to search for our species

While our results do not yet definitively demonstrate west to east routes of hominin dispersal, and the use of more detailed, high-resolution inter-stadial climate models will be required in the future to confirm their viability relative to ‘glacial’ period models, they do suggest that fieldwork and survey within the Altai Mountains, the Tian Shan Mountains, the Tarim Basin, and the Gobi Desert offer much potential to reveal Pleistocene insights into hominin dispersals. Discussions of the origins of *Homo sapiens*, the timing and tempo of human dispersal into Asia, and our species’ environmental tolerances all remain central topics in archaeology and palaeoanthropology [103]. Yet, models of dispersal into Asia have frequently focused on southern Asia and potential coastal routes [7, 26], pathways across the deserts of the Arabian Peninsula and northwestern India [10, 29], or adaptations to tropical forests in South Asia, Southeast Asia, and Melanesia [104, 105]. Our models, and evaluation of the existing data for palaeolake extents and changing central Asian temperatures, suggest that the ‘northern’ routes of dispersal represent an equally valuable geographical space for the investigation of different hominin adaptations to fluctuating aridity, high altitude, and cold temperatures, with very sensitive tempos of corridor creation and barrier constraints. We suggest that the eastern edge of the Altai Mountain chain, the Tian Shan Mountain passes, and the Gobi and Taklamakan Deserts all warrant further survey in this regard, with a view to discovering additional, sites, recovering new chronological information, and evaluating the potential fossil affiliations and timing of the arrival of different toolkits in Central, northern, and eastern Asia.

It is becoming increasingly apparent that one of the hallmarks of our species is its apparently unsurpassed ecological capacity to inhabit a number of diverse environments, across the entirety of the world’s continents, and through periods of significant climatic variability [31, 32, 34, 89]. The drivers and supporting factors behind this ecological plasticity, including cumulative culture [106–108], social networks [109], artistic or communicative expression [110, 111], demographic change [8], and technological developments [112, 113], remain up for debate. If we are to come to a better understanding of how the biological, cultural, and social evolutionary trajectories of *Homo sapiens* are intertwined, however, it is clear we need to develop high resolution palaeoenvironmental and palaeoecological models for our own species, as well as other contemporary or ancestral hominin populations, across the variety of regions they came to inhabit in the Late Pleistocene. If we are to do this effectively, our models suggest that it is not sufficient to focus purely on the ‘northern’ route of the Altai and the Transbaikal, but instead we must also examine the inner region of Central Asia, in areas previously considered to be peripheral to the Palaeolithic hominin story. We hope to have provided some geographical suggestions for future work in this regard, and highlighted frequently neglected parts of Eurasia as potentially crucial adaptive and cultural crossroads playing a significant role in the human story.

## Supporting information

S1 Fig Cumulative cost raster’s for wet route simulations. (A.) Cost-surface raster for wet routes simulation. Increasing darkness of raster has more difficult travel cost, and palaeoclimatic boundaries, such as lakes and glaciers are impassable. (B.) Cumulative cost distance raster for the wet route from the Altai Mountains. There is an obvious corridor that emerges between the Altai and Tian Shan Mountains. (C.) Cumulative cost distance raster for the wet route from the Pamir Mountains. There is a same corridor is evident between the Altai and Tian Shan Mountains (D.) Cumulative cost distance raster for the wet routes from the Tian Shan Mountains. The Tarim Basin provides the nearest corridor for dispersal.
(JPG)

S2 Fig Cumulative cost raster’s for dry route simulations. (A.) Cost-surface raster for dry routes simulation. Increasing darkness of raster has more difficult travel cost, and palaeoclimatic boundaries, such as lakes and glaciers are impassable. Arid regions (<250mm of precipitation) were given an increased cost to travel across. (B.) Cumulative cost distance raster for the dry route from the Altai Mountains. Travel through Siberia provides less costly routes, compared to across the Tarim and Dzungarian basins. (C.) Cumulative cost distance raster for the dry route from the Pamir Mountains. (D.) Cumulative cost distance raster for the dry routes from the Tian Shan Mountains.
(JPG)

S3 Fig Least cost raster results. Precise results of the least cost path analyses on a digital elevation model basemap, HYDRO1K. Red routes are from “wet” cost-surfaces and purple routes are from the “dry” cost-surface. Sites: 4. Obi-Rakhmat, 5. Shugnou, 8. Denisova, 9. Ust-Karakol, 10. Kara-Tenesh, 11. Kara-Bom, 12. Luotuoshi, 14. Gouxi, 15. Lenghu 1, 17. Chikhen Agui, 18. Tsagaan Agui, 19. Tolbor 4, 20. Kharganyn Gol 5, 21. Orkhon 1 & 7, 22. Makarovo 4, 23. Kandabaevo, 24. Varvarina Gora, 25. Tolbaga, 27. Shuidonggou 1, 28. Shuidonggou 9, 42. Yushuwan, 70. Shibazhan (75075).
(JPG)

S4 Fig Comparison of new and old Overland dispersal model. Map of the old Overland Dispersal Model (Goebel, 2015) and the new ‘Revised’ Overland Dispersal Model displayed together. The ‘Revised’ model shows a route that is more dependent on the terrain and environment of the time.
(JPG)

S5 Fig Visual compilation of palaeoenvironmental data. Map of palaeolake and palaeoclimate records throughout Northern Asia. Published extents are mapped and papers suggesting higher palaeolake levels are circled. Labels: 1. Todza Basin, 2. Khirgas Nuur, 3. Khar Us Nuur, 4. Sharga, 5. Borkhar, 6. Telmen Nuur, 7. Khovsgol, 8. Hoton Nuur, 9. Uvs Nuur, 10. Boonsagaan-Orog Nuur, 11. Boonsagaan-Orog Nuur 12–14. Boonsagaan-Orog Nuur, 15. Yabrai Salt Lake, 16. Zhuye Lake, 17. Juyanze Basin, 18. Ejina Basin, 19. Jilantai-Hetao, 20. Darhad Basin, 21. Ulaan Nuur, 22. Chuja Basin, 23. Kuja Basin, 24. Jingpeng-Dali Nor, 25. Taklamakan/Lop Nur, 26. Qaidem basin/Chaerhan Salt lake, 27. Aiding Lake, 28. Balikun Lake, 29. Manas Lake, 30. Selinco Lake, 31. Namuco Lake, 32. Tianshuihai Lake, 33. Bangongco Lake, 34. Zabuye Salt Lake, 35. Dachadan Salt Lake, 36. Qinghai Lake, 37. Daihai Lake, 38. Chaiwopu Lake, 39. Salawusu Palaeolake. The spatial extent of the used palaeoclimate records are displayed as red rectangles: I: Lake Baikal (Prokopenko et al., 2001), II. Central Asia (Li et al., 2013), III. East Asia (Li et al., 2013), IV. Kyrgyz Tien Shan (Koppes et al., 2008), V. Altai (Blomdin et al, 2018) and VI. Taklamakan Desert (Yang and Scuderi, 2008).
(PNG)

S1 Table Sites, and their citations/dating, discussed in paper. (XLSX)

S2 TableCosts for raster used in Least Cost Path analysis seen in S1 and S2 Figs.^a)^ Weighted slope costs derived from formula of: $\frac{\text{tan}\left(slope\;x{}^{\circ}\right)}{\text{tan}(1{}^{\circ})}$ (Bell and Lock, 2000). ^b)^ Aridity cost defined by Field et al. (2017) as an appropriate cost that allows short distance travels. c) NULL is the same as impermeable in GIS terms.(XLSX)

S3 Table Palaeolakes names and their citations, used for Least Cost Path analysis and maps. (XLSX)
