# Supplementary figures and images for: Heading north: Late Pleistocene environments and human dispersals in central and eastern Asia
Source: PLoS One. 2019 May 29;14(5):e0216433. doi: 10.1371/journal.pone.0216433 (PMC6541242; doi:10.1371/journal.pone.0216433)

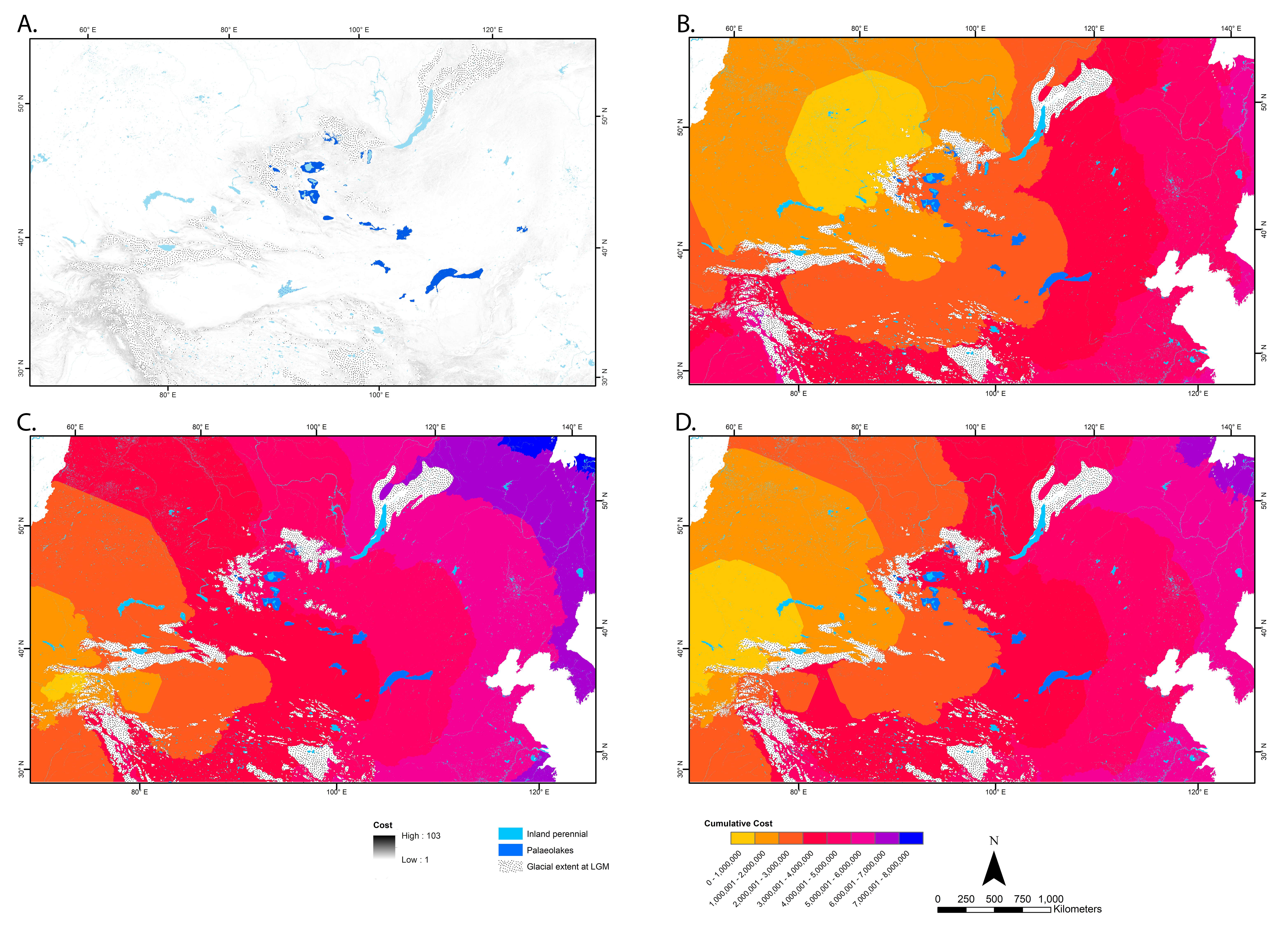

Supplement: S1 Fig — (A.) Cost-surface raster for wet routes simulation. Increasing darkness of raster has more difficult travel cost, and palaeoclimatic boundaries, such as lakes and glaciers are impassable. (B.) Cumulative cost distance raster for the wet route from the Altai Mountains. There is an obvious corridor that emerges between the Altai and Tian Shan Mountains. (C.) Cumulative cost distance raster for the wet route from the Pamir Mountains. There is a same corridor is evident between the Altai and Tian Shan Mountains (D.) Cumulative cost distance raster for the wet routes from the Tian Shan Mountains. The Tarim Basin provides the nearest corridor for dispersal. (JPG) [file pone.0216433.s001.jpg]

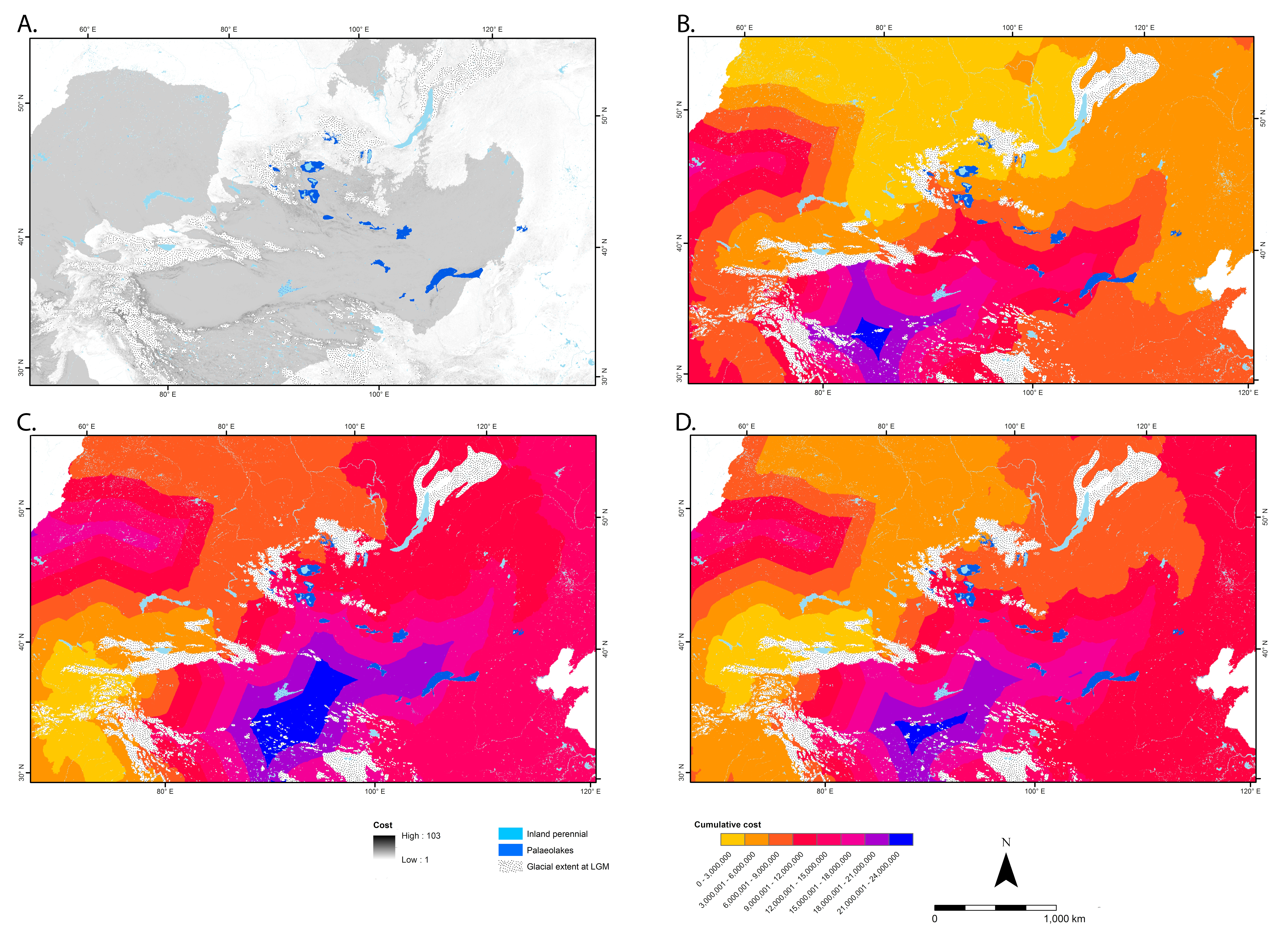

Supplement: S2 Fig — (A.) Cost-surface raster for dry routes simulation. Increasing darkness of raster has more difficult travel cost, and palaeoclimatic boundaries, such as lakes and glaciers are impassable. Arid regions (<250mm of precipitation) were given an increased cost to travel across. (B.) Cumulative cost distance raster for the dry route from the Altai Mountains. Travel through Siberia provides less costly routes, compared to across the Tarim and Dzungarian basins. (C.) Cumulative cost distance raster for the dry route from the Pamir Mountains. (D.) Cumulative cost distance raster for the dry routes from the Tian Shan Mountains. (JPG) [file pone.0216433.s002.jpg]

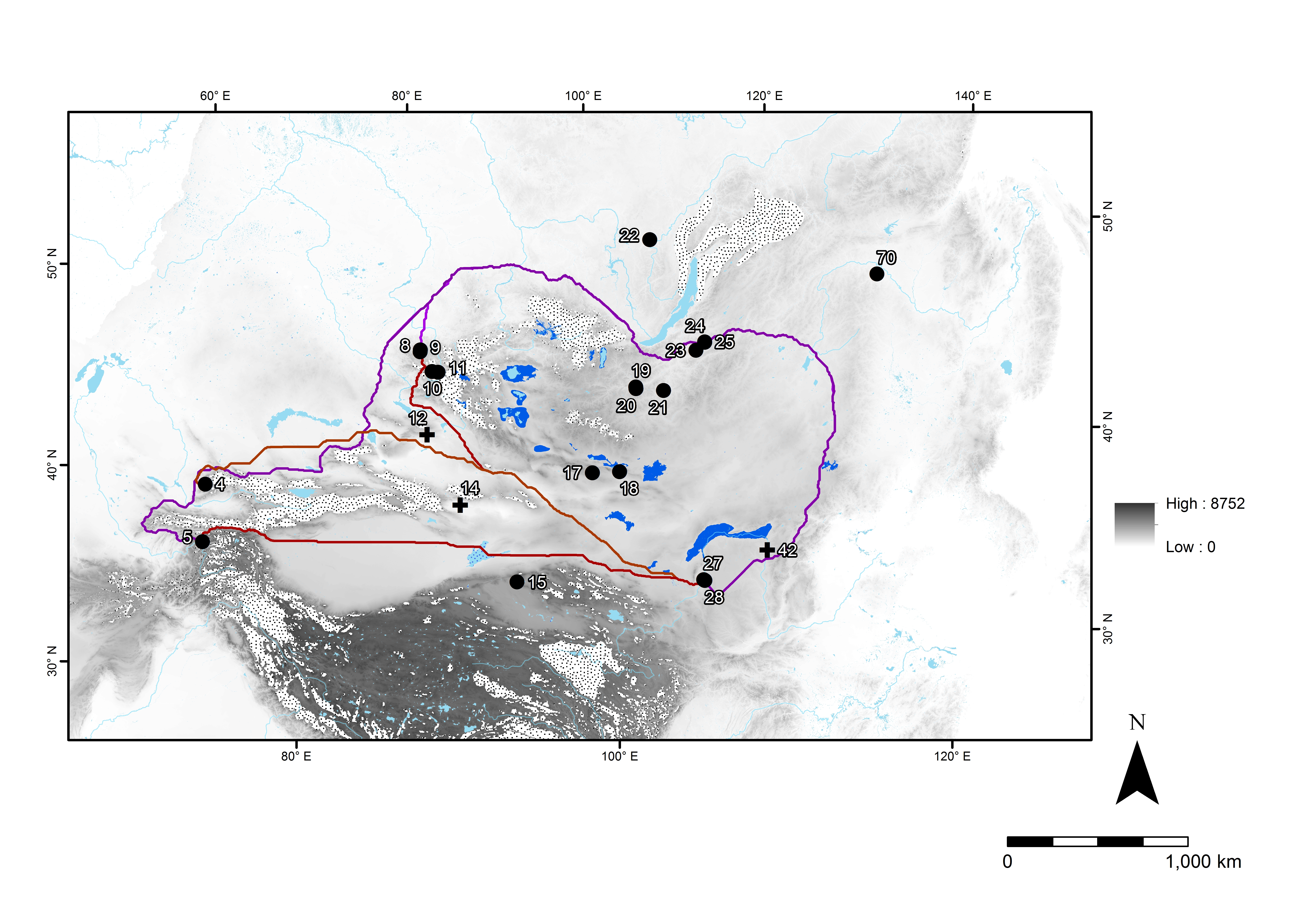

Supplement: S3 Fig — Precise results of the least cost path analyses on a digital elevation model basemap, HYDRO1K. Red routes are from “wet” cost-surfaces and purple routes are from the “dry” cost-surface. Sites: 4. Obi-Rakhmat, 5. Shugnou, 8. Denisova, 9. Ust-Karakol, 10. Kara-Tenesh, 11. Kara-Bom, 12. Luotuoshi, 14. Gouxi, 15. Lenghu 1, 17. Chikhen Agui, 18. Tsagaan Agui, 19. Tolbor 4, 20. Kharganyn Gol 5, 21. Orkhon 1 & 7, 22. Makarovo 4, 23. Kandabaevo, 24. Varvarina Gora, 25. Tolbaga, 27. Shuidonggou 1, 28. Shuidonggou 9, 42. Yushuwan, 70. Shibazhan (75075). (JPG) [file pone.0216433.s003.jpg]

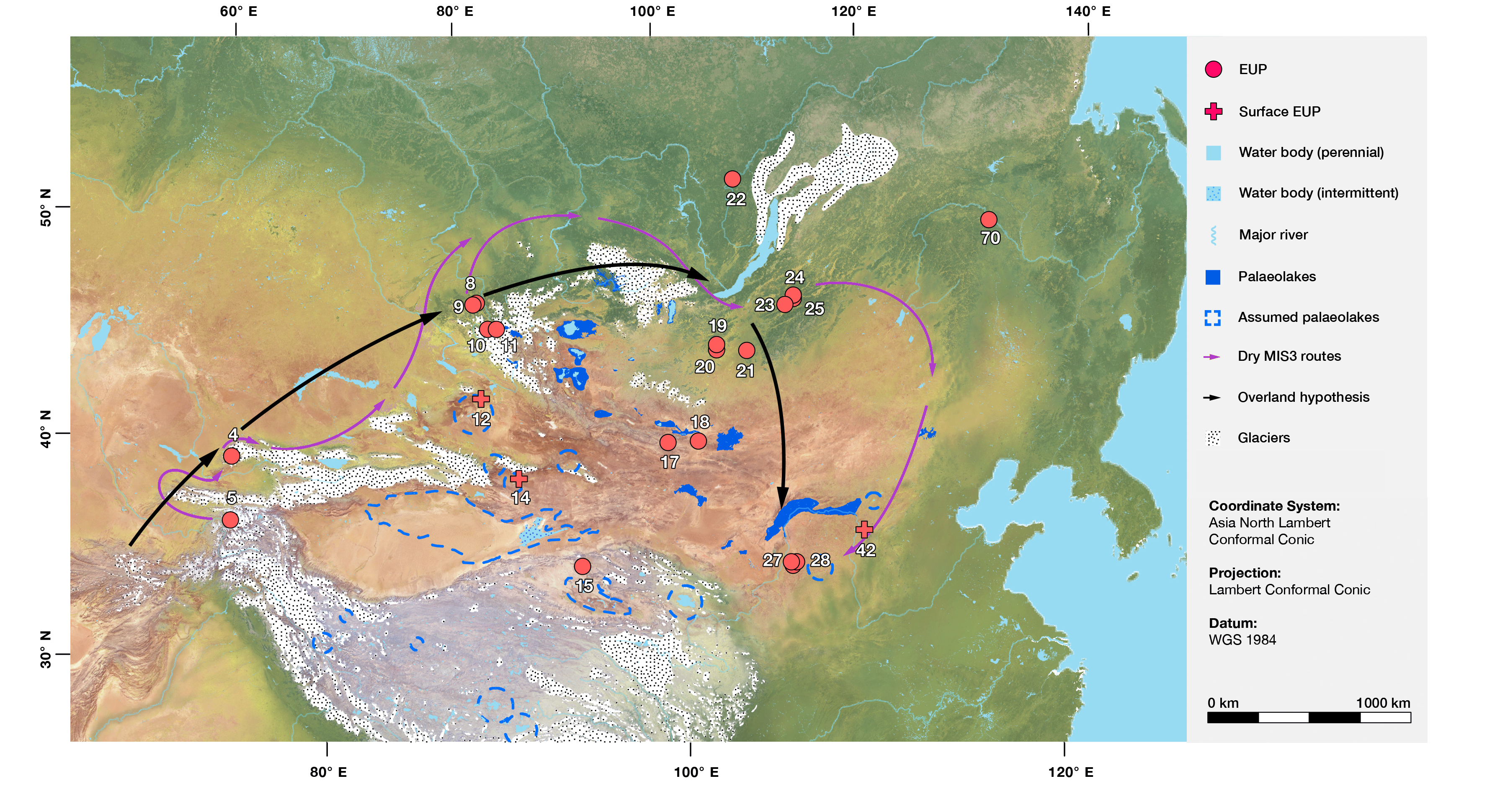

Supplement: S4 Fig — Map of the old Overland Dispersal Model (Goebel, 2015) and the new ‘Revised’ Overland Dispersal Model displayed together. The ‘Revised’ model shows a route that is more dependent on the terrain and environment of the time. (JPG) [file pone.0216433.s004.jpg]

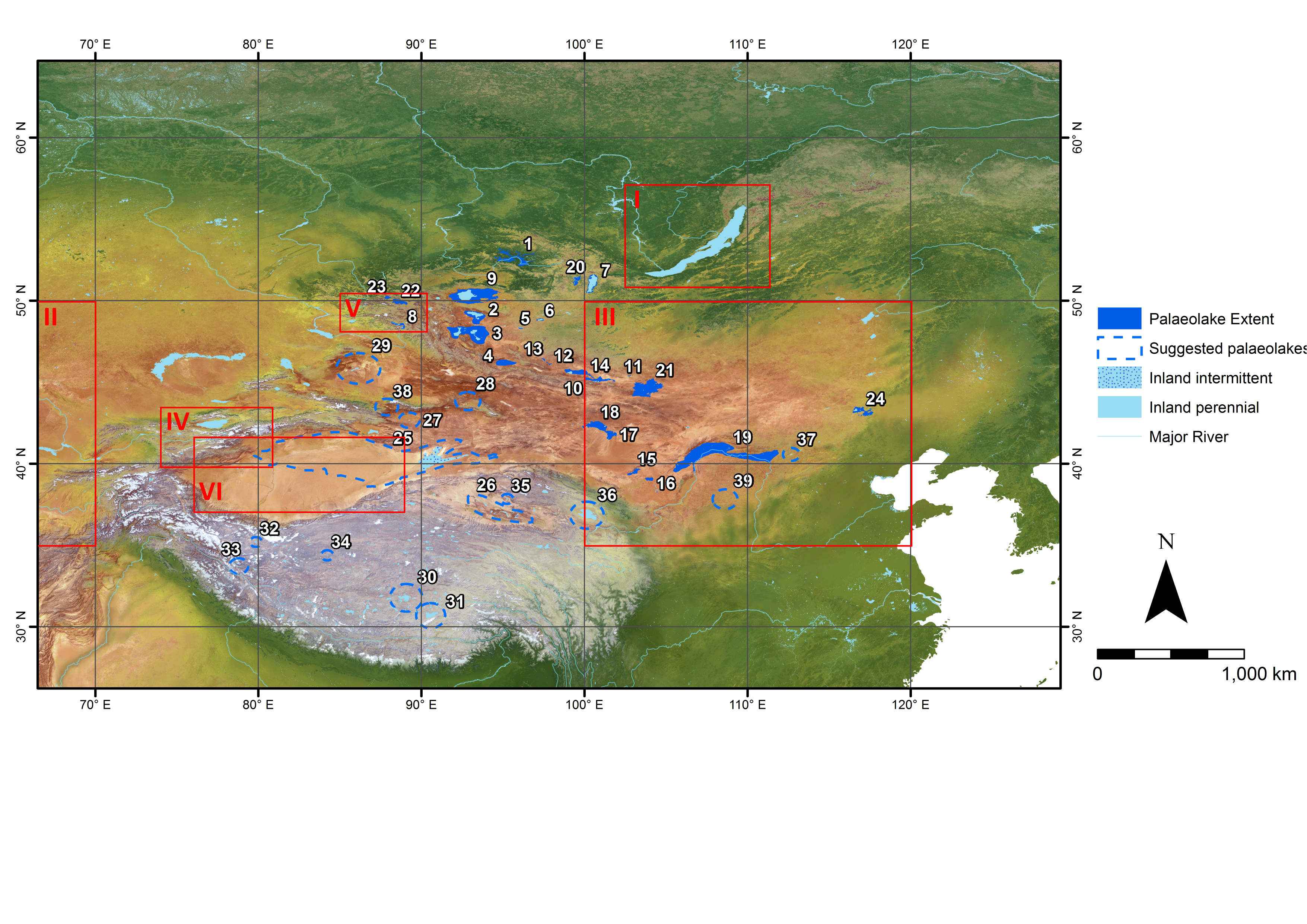

Supplement: S5 Fig — Map of palaeolake and palaeoclimate records throughout Northern Asia. Published extents are mapped and papers suggesting higher palaeolake levels are circled. Labels: 1. Todza Basin, 2. Khirgas Nuur, 3. Khar Us Nuur, 4. Sharga, 5. Borkhar, 6. Telmen Nuur, 7. Khovsgol, 8. Hoton Nuur, 9. Uvs Nuur, 10. Boonsagaan-Orog Nuur, 11. Boonsagaan-Orog Nuur 12–14. Boonsagaan-Orog Nuur, 15. Yabrai Salt Lake, 16. Zhuye Lake, 17. Juyanze Basin, 18. Ejina Basin, 19. Jilantai-Hetao, 20. Darhad Basin, 21. Ulaan Nuur, 22. Chuja Basin, 23. Kuja Basin, 24. Jingpeng-Dali Nor, 25. Taklamakan/Lop Nur, 26. Qaidem basin/Chaerhan Salt lake, 27. Aiding Lake, 28. Balikun Lake, 29. Manas Lake, 30. Selinco Lake, 31. Namuco Lake, 32. Tianshuihai Lake, 33. Bangongco Lake, 34. Zabuye Salt Lake, 35. Dachadan Salt Lake, 36. Qinghai Lake, 37. Daihai Lake, 38. Chaiwopu Lake, 39. Salawusu Palaeolake. The spatial extent of the used palaeoclimate records are displayed as red rectangles: I: Lake Baikal (Prokopenko et al., 2001), II. Central Asia (Li et al., 2013), III. East Asia (Li et al., 2013), IV. Kyrgyz Tien Shan (Koppes et al., 2008), V. Altai (Blomdin et al, 2018) and VI. Taklamakan Desert (Yang and Scuderi, 2008). (PNG) [file pone.0216433.s005.png]
